# *Clostridioides difficile* as a Dynamic Vehicle for the Dissemination of Antimicrobial-Resistance Determinants: Review and In Silico Analysis

**DOI:** 10.3390/microorganisms9071383

**Published:** 2021-06-25

**Authors:** Philip Kartalidis, Anargyros Skoulakis, Katerina Tsilipounidaki, Zoi Florou, Efthymia Petinaki, George C. Fthenakis

**Affiliations:** 1 Department of Clinical and Laboratory Research, Faculty of Medicine, School of Health Sciences, University of Thessaly, 41110 Larissa, Greece; kartalidisphilip@gmail.com (P.K.); skulakis@gmail.com (A.S.); tsilipoukat@gmail.com (K.T.); zoi_fl@hotmail.com (Z.F.); petinaki@uth.gr (E.P.); 2 Veterinary Faculty, University of Thessaly, 43100 Karditsa, Greece

**Keywords:** antimicrobial resistance, *Clostridioides difficile*, gene, genome, in silico analysis, transposon, vancomycin

## Abstract

The present paper is divided into two parts. The first part focuses on the role of *Clostridioides difficile* in the accumulation of genes associated with antimicrobial resistance and then the transmission of them to other pathogenic bacteria occupying the same human intestinal niche. The second part describes an in silico analysis of the genomes of *C. difficile* available in GenBank, with regard to the presence of mobile genetic elements and antimicrobial resistance genes. The diversity of the *C. difficile* genome is discussed, and the current status of resistance of the organisms to various antimicrobial agents is reviewed. The role of transposons associated with antimicrobial resistance is appraised; the importance of plasmids associated with antimicrobial resistance is discussed, and the significance of bacteriophages as a potential shuttle for antimicrobial resistance genes is presented. In the in silico study, 1101 *C. difficile* genomes were found to harbor mobile genetic elements; Tn*6009*, Tn*6105*, CTn*7* and Tn*6192*, Tn*6194* and IS*256* were the ones more frequently identified. The genes most commonly harbored therein were: *ermB*, *blaCDD*, *vanT*, *vanR*, *vanG* and *vanS*. Tn*6194* was likely associated with resistance to erythromycin, Tn*6192* and CTn*7* with resistance to the β-lactams and vancomycin, IS*256* with resistance to aminoglycoside and Tn*6105* to vancomycin.

## 1. Background

### 1.1. Introduction

*Clostridioides difficile* is a Gram-positive, anaerobic, spore-forming, toxin-producing organism, which was officially renamed in 2016 from *Clostridium difficile* [1]. The pathogen is responsible for healthcare-associated diarrhea; in recent years, the microorganism has been also incriminated in community-associated diarrhea [2].

A broad spectrum of clinical manifestations has been reported in cases of infection. These range from mild to serious diarrhea, to pseudomembranous colitis and fulminant colitis, which are life-threatening. Various factors are considered to increase a person’s risk of infection with the organism; these include a prolonged stay in healthcare settings, exposure to broad-spectrum antibiotics disrupting the microbiome of the patients, advanced age, underlying diseases, the use of drugs that act as proton pump inhibitors and various immunocompromising conditions. It is also notable that, in a significant proportion of patients (20–30%), a recurrent episode of the infection occurs [2].

*C. difficile* infection mostly occurs as the result of the ingestion of spores, combined with an abnormal or disrupted colonic mucosa. Spores of a toxigenic strain can enter into a patient through personal contact or the environment. Although gastric acid in the intestine is a generally effective barrier to many pathogens, *C. difficile* spores can colonize the intestine, which would trigger the production of two toxins from vegetative *C. difficile*. These toxins are: enterotoxin TcdA and cytotoxin TcdB, which are encoded by the *tcdA* and *tcdB* genes, respectively. In several strains of the organisms, two genes, *cdtA* (enzymatic component) and *cdtB* (binding component), are expressed, producing a possible virulence factor. While it is not yet known if the toxin contributes to the pathogenicity of *C. difficile*, the first strain that was found to express the binary toxin gene caused severe pseudomembranous colitis [3].

In host cells, receptor binding and internalization take place, and the toxins’ functions are exerted mainly through glycosylating and thus, disrupting various intracellular signaling pathways, the regulation of which is mediated by the Rho family of small GTPases (a detailed list of abbreviations used in the manuscript is in Appendix A). As toxins TcdA and TcdB modify normal cellular activity, a disruption of colon mucosa can be observed, along with the secretion of a variety of pro-inflammatory cytokines and the apoptosis of colonic epithelial cells. In response to these ongoing effects, PMN are recruited to the site, a major characteristic of one the most severe forms of CDI, pseudomembranous colitis [2]. Although toxins are responsible for various symptoms of the infection, their relative levels in feces do not seem to reflect the level of severity of a particular case of the infection [4]. Natural antibodies against toxins A and B are important; asymptomatic carriers have high concentrations of them and do not develop diarrhea [2].

*C. difficile* infects mostly infants (15–70%), although adults may also be infected and develop clinical disease. The risk of colonization is considerably higher in hospital environments and nursing homes [2]. Asymptomatic carriers, infected patients, contaminated environments and animal intestinal tracts are all potential reservoirs for *C. difficile*. Spores play an important role in the dissemination of the microorganism, as they are resistant to heat, acid and antimicrobials, and they can survive in the environment for several months [5]. On the other hand, spores may germinate and cause a relapse of CDI after the cessation of therapy.

The administration of the following antimicrobial agents has been associated with the development of CDI: ampicillin, amoxicillin, cephalosporins, clindamycin and fluoroquinolones, as these disrupt the normal intestinal microbiota and allow for the proliferation of *C. difficile*. Metronidazole, vancomycin and fidaxomicin are antimicrobials to which *C. difficile* is susceptible; hence, they are effective in the treatment of primary and recurrent CDI [6]. With regard to the type of the antimicrobial to be administered, the severity of CDI is taken into consideration, based on the recommendations of the Infectious Disease Society of America and the European Society of Clinical Microbiology and Infectious Diseases.

Currently, *C. difficile* isolates with multiple-antibiotic resistance have emerged, such as the hyper-virulent ribotypes O27 and O78 strains, a phenomenon that is becoming a heavy burden in CDI treatment [7,8]. Further, the ability of *C. difficile* to form spores and their subsequent persistence in adverse environments may also have a role in the acquisition of novel antibiotic-resistance determinants. Additionally, biofilm formation is another characteristic of *C. difficile* that possibly contributes to antimicrobial resistance, although its precise role in the acquisition of resistant determinants has not yet been fully clarified.

In the last two decades, PCR and sequence-based techniques, particularly WGS, have significantly furthered our knowledge of the genetic diversity, evolution, epidemiology, and pathogenicity of *C. difficile* [9]. *C. difficile* is defined by a large diverse pan-genome, with extreme levels of evolutionary plasticity that has been shaped over long periods by gene flux and recombination, often between divergent lineages. These evolutionary events are in response to environmental and anthropogenic activities and have led to the rapid emergence and worldwide dissemination of virulent and resistant clonal lineages [9].

The present paper is divided in two parts. The first part focuses on the role of *C. difficile* in accumulating genes associated with antimicrobial resistance and then, transmitting them to other pathogenic bacteria that occupy the same human intestinal niche. The second part describes an in silico analysis of the genomes of *C. difficile* available in GenBank, with regard to the presence of mobile genetic elements and antimicrobial resistance genes.

### 1.2. The Diversity of Clostridioides difficile Genome

Several genomes of *C. difficile,* ranging in size from 4.1 to 4.3 Mb, have been fully sequenced. The pan-genome of *C. difficile* is comprised of a core genome and an accessory (adaptive) genome. *C. difficile*’s “open” genome is characterized by its plasticity, with access to and frequent exchange with multiple host environments and bacterial gene pools.

*C. difficile* has a highly dynamic and mosaic genome, comprising a high proportion (∼11% in some cases) of MGEs. These include bacteriophages, group I introns, insertion sequences, *sigK* intervening elements, CRISPR-*cas* elements, genomic islands and transposable and conjugative elements, accompanied by an extensive range of accessory genes [10,11,12,13,14]. These are responsible for the remarkable genetic diversity in the *C. difficile* genome, due to the acquisition of foreign DNA coding for ecological adaptations and the novel resistant/virulent phenotypes seen in this species.

A large portion of the genetic elements found in *C. difficile*’s genome are transposable and have the ability to change their position within the genome. The *C. difficile* genome possesses both mobilizable and self-transmissible transposons [15,16]. The former requires complex host mechanisms in order to change their positions; the latter, also known as conjugative transposons or ICEs, are able to move independently. Through the expression of the enzymes of integrase, excisionase and, in some cases, a site-specific recombinase, CTns can be excised, transferred and integrated into the *C. difficile* genome, as well as into bacteria of other species/genera [17]. Consequently, the presence of MGEs, and especially transposons, in *C. difficile*’s pan-genome plays a pivotal role in the acquisition of *C. difficile*’s genetic elements from various bacteria, as well as in the insertion of genes into *C. difficile*’s genome from the intestinal metagenome [18].

Exposure to antimicrobials plays a significant role in the pathogenesis of CDI, and resistance should be considered a virulence factor. Resistance to antimicrobials in *C. difficile* is mediated predominantly by Tns [10,16,18]. Currently, with the help of techniques such as PCR and studies of WGS, various Tns have been defined that are associated with antimicrobial resistance [8,9,19]. *C. difficile* has an abundance of plasmids that encode functions potentially relevant to the pathogenesis of CDI. However, only a limited number of them have been associated with antimicrobial resistance, and it is unknown whether they are transferable [20].

### 1.3. The Current Status of Resistance of Clostridioides difficile to Various Antimicrobial Agents

Antimicrobial resistance in *C. difficile* may be the consequence of multiple mechanisms [21,22]. Such factors and pathways include the resistance-associated genes carried in the bacterial chromosome, alterations in the site-specific targets of antibiotics and/or in metabolic pathways in *C. difficile*, mobile genetic elements, mutations and biofilm formation.

A broad spectrum of antimicrobial-resistance genes has been identified in the *C. difficile* genome, conferring resistance to various classes of antibiotics. The *C. difficile* genome harbors a broad spectrum of relevant genes and mutations that can lead to resistance to various classes of antibiotics [21]. Alterations in housekeeping genes have also been described for uncommonly used antibiotics in CDI treatment, probably as the result of in vivo selective pressure resulting in the development of antibiotic-resistance phenotypes. This phenomenon has been observed in cases of using fluoroquinolones and antibiotics belonging to the rifamycin class. This has also been observed with antibiotics of the rifamycin class of antibiotics, with resistance resulting from mutations in the *β* subunit of bacterial RNA polymerase, *rpoB*.

Today, resistance in *C. difficile* is observed for multiple antibiotics, including aminoglycosides, lincomycin, erythromycin, penicillins, tetracyclines, clindamycin, cephalosporins and fluoroquinolones, all of which are commonly used in the treatment of bacterial infections in clinical settings. Recent studies (2012 to 2015) have revealed that the antimicrobial agents to which *C. difficile* isolates are more commonly resistant include clindamycin (8.3–100%), cephalosporins (51%), erythromycin (13–100%) and fluoroquinolones (47%) [8].

A noteworthy point is that resistance rates depend on various factors, for examples, geographical locations and policies on the usage of antimicrobial agents [7]. Moreover, the breakpoints for defining resistance during laboratory testing differ between the Clinical & Laboratory Standards Institute and the European Committee on Antimicrobial Susceptibility Testing for some antimicrobials (e.g., vancomycin), which can affect the interpretation of results. Comparative evaluation of data from studies in countries using the one or the other system may lead to unclear results due to the use of different standards.

## 2. The Role of the Mobile Genetic Elements of *Clostridioides difficile* in the Acquisition and Transmission of Antimicrobial Resistance

*C. difficile* may acquire accessory AMR genes, many of which can be horizontally transferred both within and between species. Various mobile genetic elements (transposons, plasmids, bacteriophages) have been identified in this species and can be carriers of specific AMR genes.

### 2.1. Transposons Associated with Antimicrobial Resistance

#### 2.1.1. MLS_B_ Antibiotics

The main mechanism of resistance to MLS_B_ is ribosomal methylation due to erythromycin ribosomal methylases encoded by the *erm* genes (*ermA, ermB, ermC,* etc.). The main AMR gene for *C. difficile*’s resistance to erythromycin is *erm*(B). This is mostly found in the non-conjugative and self-transmissible Tns Tn*6194,* Tn*5398,* Tn6218 and Tn*6215* [23].

Tn*5398* is a mobilizable non-conjugative element, 9.6 kb in length, containing two identical copies of *erm*B genes, separated by a copy of the direct repeat sequence that is found on either side of the *ermB* gene in the *Clostridium perfringens* genome. Tn*5398* lacks the genes for recombinase and, as a result, its integration to the recipient strain depends on other CTns in the donor’s genome. This element is transferred in vitro from *C. difficile* to *Staphylococcus aureus* and to *Bacillus subtilis* [24,25]. Tn*5398* with the duplicated *erm*(B) gene was first described in strain *C. difficile* 630, which was originally recovered in Switzerland; later, in 2001, Farrow et al. [26] found the same organization in isolates of the organism recovered in Australia and France, providing evidence that this element can move freely between *C. difficile* isolates.

Tn*6194* is a CTn first identified in *C. difficile* 2,007,855 [27,28]. Its conjugative region presents similarity with that of Tn*916,* a large family of conjugative elements widely spread in Gram-positive and Gram-negative bacteria, while its accessory region resembles the one of Tn*5398*; this modular composition is commonly observed amongst the MGEs in *C. difficile*. The transfer of Tn*6194* from *C. difficile* to *Enterococcus faecalis* has occurred in vitro [28]. Tn*6215* is another mobilizable transposon, with a length of 13 kb. It has been found to be transferred among *C. difficile* strains by a conjugation-like mechanism or by phage ΦC2 transduction [29,30]. Finally, Tn*6189*, described for the first time in 2019, a carrier of *erm*(B) gene, was found in a collection of both non-toxigenic and toxigenic *C. difficile* isolates recovered in Southeast Asia, supporting the idea of intraspecies horizontal transfer [31]. Besides those three Tns, a novel Tn*916*-like transposon, similar to Tn*6218*, can also contribute to resistance to MLS_B_ antibiotics. This element, as described below, can participate in the transfer of the *cfr* gene, which provides resistance to lincosamides, especially when the *erm* genes are not present [32].

Furthermore, in a multidrug-resistant clone from PCR ribotype 017 *C. difficile,* a new alleged mobile element of mosaic structure and 61.3 kb in length was found, presenting ermG, *mefA*/*msrD* and *vat* genes that contribute to the macrolide, lincosamide and streptogramins resistance [33]. This element has homology with other non-*C. difficile* genomes, whereas the cluster of resistance genes is found in many bacterial species, mostly in *Enterococcus cecorum*, *Streptococcus* spp., *Neisseria gonorrhoeae* and *Acinetobacter junii,* indicating the possibility of interspecies transmission.

#### 2.1.2. Tetracyclines

The acquisition of tetracycline resistance in *C. difficile* strains is generally associated with Tn*5397*, Tn*916* or the Tn-*916*-like family and Tn*6164* transposons. It has been observed that these mobile elements can transfer the *tet* class of genes, including *tetM*, *tetW* and *tet44*. *TetM* is the most common gene of the *tet* class, while the transposons that harbor it most frequently are Tn*5397,* Tn*916* or the Tn-*916-*like family [8,34].

Tn*5397* is the transposon that is best described and understood among the *C. difficile* conjugative Tns. First identified in strain 630, it is an element 21 kb in size that closely resembles Tn*916,* which is the paradigm of this MGE family. It can be transferred between *C. difficile* strains and between *B. subtilis* and *Enterococcus faecalis* and has also been identified in *Streptococcus* spp. Tn*5397* includes a large serine recombinase, TndX, responsible for integration and excision that directs the element into two highly preferred insertion sites in *C. difficile* R20291. It is noteworthy that the two ends of Tn*5397* differ from each other [35].

Tn*916* is another mobile element associated with tetracycline resistance, which was transferred and received from B. subtilis. Tn*916,* unlike Tn*5397*, in addition to a tyrosine recombinase, also contains an excisionase; both are responsible for the integration and excision of the element and provide Tn*916* with the ability to insert at multiple sites into the *C. difficile* genome, although it has a preferred consensus site, which is present in the *C. difficile* genome in 10^5^ times [35].

Furthermore, another two mobile elements, *Tn6190* and *Tn6235*, both carriers of the *tetM* gene, have been identified in a collection of *C. difficile* strains, recovered from a diversity of hosts (humans and pigs) [36]. The mechanism by which *C. difficile* acquires the *tetM* gene remains unclear. A proposed model is that the organism acquires this gene through genetic transfer from other bacteria containing *tetM*, e.g., *Bifidobacterium longum.* The *tetW* has the second largest host range and is associated with conjugative and non-conjugative elements that may vary among different bacterial species. The gene was a new allele, indicating 99% sequence identity to the gene found in the human *B. longum* F8 strain, thus supporting the hypothesis of horizontal gene transfer events in the rapid diffusion of this tetracycline-resistance determinant [37]. The *tet44* gene was found to be carried by Tn*6164* in some *C. difficile* RT078 isolates; this putative conjugative Tn also contains aminoglycoside resistance genes, e.g., *ant9(Ia) and ant(6)* [38]. In reference strain MT20, Tn*6164* is located in a novel 106 Kb genetic island that is made up mainly of mobile elements from non-clostridial species, including *Streptococcus pneumoniae*, *E. faecalis* and *Thermoanaerobacter* spp.

#### 2.1.3. Vancomycin

For almost 30 years, vancomycin has been effectively employed in the first-line treatment of CDI, including cases caused by isolates within epidemic lineages and with increased resistance to metronidazole [6]. Until now, there were no known underlying mechanisms for vancomycin resistance, despite several reports of increased resistance to this antibiotic. Genome sequencing studies in *C. difficile* have revealed that a vancomycin resistance operon (*vanG_Cd_*) was present, which contained a cryptic gene cluster with homology to the *vanG*-type operon of *E. faecalis* BM4518 [39]. When present, this is found at the same genomic location in all *C. difficile* strains; although it is often referred to as cryptic (phenotypically silent), transcriptional and biochemical studies have shown that this cluster acts as an operon that contributes slightly to an increase in inhibitory concentration in *C. difficile* (from 1 to 2 mg L^−1^) [40]. It is also noteworthy that a report of a ~42 kb element showed significant homology to Tn*1549*, a conjugative Tn associated with the emergence and global dissemination of vancomycin-resistant enterococci, that contained a cryptic *vanB2* operon [41].

#### 2.1.4. Linezolid

The multiresistance-associated *cfr* gene (providing resistance to oxazolidinones, chloramphenicol, lincosamides, pleuromutilins and streptogramin A) was first discovered in a *Staphylococcus sciuri* plasmid; its mechanism of action relies on the methylation of position A2503 of the 23S rRNA gene by a rRNA methyltransferase [42,43]. This gene was found to be present mainly in transferable plasmids and is disseminated in close to 20 different genetic contexts of isolates of various bacterial species, e.g., *Enterococcus* spp., *Bacillus* spp., *Proteus vulgaris*, *Escherichia coli*, *Macrococcus caseolyticus*, *Jeotgalicoccus pinnipedialis* and *Streptococcus suis,* recovered in a broad geographic diversity, including Europe, Latin America, the United States and Asia [44,45].

Two *cfr*-like genes are usually found in *C.* *difficile,* *cfr*(B) and *cfr*(C), with protein sequence similarity to Cfr being less than 80% [44]. The *cfr*(B) was first located in strain 11,140,508, contained within Tn*6218*-like elements [46,47]. As for *cfr*(C), after Candela et al. [48] defined it in the *C. difficile* T10 strain, it has been detected in three types of ICEs in various strains, including the non-toxigenic strain *C. difficile* F548 [48]. Further, a new *cfr*(E) gene was discovered in several *C. difficile* isolates recovered in Latin America [49]. Finally, it is important to note that the location of *cfr-*genes in conjugative transposons may play a role in the ease of their transfer.

#### 2.1.5. Chloramphenicol

Chloramphenicol resistance in *C. difficile* is mediated by the closely related *catD* and *catP* genes that encode chloramphenicol acetyltransferases, as evidenced by hybridization studies [50,51,52]. Tn*4453*a and Tn*4453*b are transposons, in which *catP* gene is harbored, that are almost identical to each other; they were first discovered in *C. difficile* strain W1 and present functional and structural similarity with the Tn*4451* mobilizable element found in Clostridium perfringens [52]. In contrast to *catP*, the *catD* gene appears to be present in at least two copies on the *C. difficile* chromosome [50]. It is not yet known whether *catD* can transfer either within or between species [51].

### 2.2. Extra-Chromosomal Elements (Plasmids) Associated with Antimicrobial Resistance 

Extra-chromosomal elements, such as plasmids, are significant in most bacterial species. Until today, in the case of *C. difficile*, there has not been any systematic identification of plasmids, while the few that have been characterized do not seem to contribute to antimicrobial resistance. According to an in silico analysis based on publicly available sequence data, only about 13% of total *C. difficile* strains contained ECEs, with the relative number of elements varying from 1 to 6. ECEs have been classified into six homology groups, based on their sequence similarity to a group of singleton ECEs that were identified [20].

In the study of Hornung et al. [20], a circular plasmid of 6.2 kb in length, named pCD-SMR, was identified, carrying a gene that encoded a TetTR-family regulator, close to a small multidrug-resistance protein [20]. Although these functions are not very specific and are common even in non-AMR-related locations, the mentioned transporter presents similarity to the multidrug efflux transporter *EbrB* found in *B. subtilis* (UniProt P0CW82; 46% identity over 89% of the *C. difficile* protein length), as well as with the multidrug efflux transporter *EmrE* of *E. coli* (UniProt P23895; 40% identity over 90% of the *C. difficile* protein length). Apart from the TetTR-family, regulator pCD-SMR contained a MobA/MobL mobilization protein (IPR005053; ORF8) and a winged helix-like DNA-binding domain superfamily protein (IPR036388; ORF3). Part of the drug transporter and the regulator, a genomic content of about 1 kb, might have originated from plasmid pK5 of an unidentified organism (KJ792090.1) or a *Listeria* transposon.

A single ECE with an aminoglycoside-2’’-adenylyltransferase motif and high homology to a lincosamide-resistance protein from *Staphylococcus haemolyticus* (UniProt P06107; 55% identity over 99% of the *C. difficile* protein length) has been also identified. The assembly of the ECE was comprised of nine contigs, with a total size of 5384 bp, showing a high percentage of identity with *Campylobacter jejuni*’s various genomic and plasmid regions [20].

It is shown that most plasmids that are part of either the ECE4 or ECE6 family seem to acquire the genes *bla*_R1_ (IPR008756) and *bla*_I_. Although they do not contribute to antibiotic resistance, they are associated with the regulation of *β*-lactamases induction in various species, e.g., *Staphylococcus* spp., as well as *C. difficile* [53].

While strains with reduced susceptibility to metronidazole have been recovered in various parts of the world, the exact mechanism of reduced susceptibility to metronidazole in *C. difficile* isolates has not yet been elucidated. Recently, a plasmid linked to metronidazole resistance in *C. difficile* was identified. The pCD-METRO plasmid, which has spread internationally, is defined by its high copy number and is disseminated in diverse PCR ribotypes, including those known to cause outbreaks, a case of a plasmid for which evidence of possible horizontal transmission is provided [54].

### 2.3. Bacteriophages as a Potential Viral Shuttle Carrying Antimicrobial Resistance Genes

Phages have been recognized to be essential actors in various aspects of bacterial ecology, e.g., bacterial population regulation, and particularly in gene transfer between bacteria, including antibiotic-resistance genes. Various phages have been identified in *C. difficile* isolates, e.g., ϕCD119, ϕCD38-2 and ϕCD27, which have been associated with the regulation of toxin production.

Among the various phages identified in *C. difficile*, ΦC2 is considered to be capable of transferring genes conferring resistance to MLS antibiotics; such genes are located on the Tn6215 transposon. This shows the importance of phages in the transduction of various genes, including antimicrobial-resistance genes [35].

### 2.4. The Role of Clostridioides difficile as a Reservoir for the Dissemination of Antimicrobial Resistance Determinants

Bacteria can evolve at a very rapid pace, as the results of their horizontal gene transfer ability contributes to their high levels of genome plasticity. This characteristic is also responsible for the problem of antibiotic resistance. Horizontal transfer can happen through transformation, transduction or conjugation. Transformation is the process during which the naked DNA is uptaken by the recipient organism; during transduction, the transfer of bacterial DNA is achieved by phages; finally, during conjugation, the transfer between the donor and the recipient organisms is achieved through various genetic elements that contain the DNA [55,56,57].

*C. difficile* shows significant similarities in the sequence of determinants associated with the development of antibiotic resistance with various other pathogens. This indicates that *C. difficile* can be used as a dynamic vehicle. While the organism is naturally resistant to aminoglycosides, some strains contain genes that confer resistance to aminoglycosides can spread from other bacteria, as with *aadE* and *aac(6′)-Ie-aph(2″)-Ia* aminoglycoside-modifying enzymes, which can be found commonly in *Enterococcus* spp., *Campylobacter* spp., *Staphylococcus spp*. or *Streptococcus* spp. [58]. Although the presence of such genes should not necessarily be correlated with aminoglycoside resistance in anaerobe bacteria like *C. difficile*, their contribution to aminoglycoside resistance through being spread to other species is important. Another example of *C. difficile* acting as a dynamic gene vehicle is the case of *npmA*, a gene originally found in a clinical *E. coli* isolate that provided pan-aminoglycoside resistance by means of encoding an uncommon 16S rRNA methyltransferase. This gene was also found in *C. difficile*’s genome, specifically within open reading frames that are associated with recombination; this suggests that it was acquired via horizontal gene transfer, which can be assumed, taking into consideration the mosaic structure of *C. difficile* [58].

*C. difficile* can acquire genes encoding class D–*β*-lactamases (CDD), which are intrinsic to this species [59]. Both CDD-1 and CDD-2 genes confer resistance to a wide variety of *β* lactam antibiotics, including penicillins and expanded-spectrum cephalosporins. It has been suggested that, as these genes are not adjacent to any MGE-related ones, mobile elements do not contain them; moreover, while the promoters of these genes allow for only a limited expression, when they were controlled by efficient promoters in an antibiotic-sensitive *Clostridium cochlearium* strain, high-level resistance to –*β*-lactams was observed [59]. When considered cumulatively, the results indicate the existence of intrinsic class D–*β* lactamases in *C. difficile*, which constitute a reservoir of highly potent enzymes capable of conferring broad-spectrum, clinically relevant levels of resistance to –*β*-lactam antibiotics.

Overall, horizontal genetic transfer events can play an important role in the evolution of *C. difficile* and point to its potential as a resistance reservoir in the human gut [26,60,61]. An organism harboring such a relevant number of antimicrobial-resistance determinants in mobile elements may likely trigger a dissemination of these determinants intra- and interspecies. Antibiotic selective pressure promotes horizontal genetic transfer in *C. difficile*. *C. difficile* may significantly contribute to the spread of antibiotic-resistance determinants to other bacterial genera and species that share the same environmental or host niche.

## 3. In Silico Analysis of 2190 *Clostridioides difficile* Genomes

### 3.1. Procedures

As part of this study, an in silico analysis was carried out. The genomes of all *C. difficile* strains available in the GenBank database were downloaded. A total of 2190 genomes were found to be eligible and used for the analysis. The selection of the eligible genomes was done by a Perl script, using the following criteria: the assembly level of the genome in National Center for Biotechnology Information (Bethesda, MD, USA) was complete or the N50 of the assembly of the genome was above 100,000. Subsequently, the 2190 genomes of *C. difficile* strains were analyzed using the MobileElementFinder tool (ver.1.0.3) (Center for Genomic Epidemiology, Technical University of Denmark, Lyngby, Denmark), in order to find the various MGEs. Finally, after the characterization of MGEs in each genome, MGEs were searched for the presence of AMR genes using the tool AMRFinderPlus (ver. 3.9) (National Center for Biotechnology Information).

### 3.2. Findings

A total of 1101 genomes (50.3% of those examined) were found to harbor at least one mobile genetic element. In total, 32 MGEs were found in all of these genomes. The most commonly identified transposons were Tn*6009*, Tn*6105*, CTn*7*, Tn*6192*, Tn*6194* and IS*256*, respectively (Table 1). The MGEs in 533 (48.4%) genomes harbored antimicrobial-resistance genes (Table 1). The ranking of these MGEs was modified when the presence of antimicrobial-resistance genes was considered therein. For example, Tn*6009* was present in 408 genomes, but only in 71 of these (17.4%) did it harbor AMR genes; in contrast, Tn*6194* was present in 108 genomes, of which in 104 (96.3%) it harbored AMR genes.

The analysis indicated that the genes most commonly (>10% of all genes) harbored in MGEs were: *ermB*, *blaCDD*, *vanT*, *vanR*, *vanG* and *vanS* (Table 2). Tn*6194* seemed to be particularly associated with resistance to erythromycin, as in all cases it harbored *erm(B)*. Tn*6192* and CTn*7* seemed to confer resistance to the *β*-lactams and vancomycin. IS*256* appeared to contribute mostly to resistance to aminoglycoside, and Tn*6105* was associated with resistance to vancomycin. Details are in Table 3.

The analysis indicated that the genes most commonly (>10% of all genes) harbored in MGEs were: *ermB*, *blaCDD*, *vanT*, *vanR*, *vanG* and *vanS* (Table 2). Tn*6194* seemed to be particularly associated with resistance to erythromycin, as in all cases it harbored *erm*(B*)*. Tn*6192* and CTn*7* seemed to confer resistance to the *β*-lactams and vancomycin. IS*256* appeared to contribute mostly to resistance to aminoglycoside, and Tn*6105* was associated with resistance to vancomycin.

## 4. Conclusions

The role of *C. difficile* in the dissemination of antimicrobial resistance has been appraised. The organism possesses multiple mechanisms, through which it can acquire and transmit antimicrobial-resistance genes.

Bacterial spores can act as recipients of antimicrobial-resistance genes. *C. difficile* spores are widely distributed in the environment. The environmental origin of antimicrobial-resistance genes is not a hypothetical situation, as many such genes originate from the environment and persist in reservoirs there. Studies conducted in many environmental matrices have supported the hypothesis that non-impacted/pristine venues and impacted environments can become a source of such genes, ending in bacteria within the agro-food system and finally, in pathogenic bacteria. Hence, the ecology of the organism enhances its potential to acquire antimicrobial-resistance genes. The first *C. difficile* isolates resistant to macrolides, lincosamides and tetracycline were described in 1980. These antibiotics are frequently used for therapeutic purposes in animal production and are excreted practically unaltered to the environment, generating a pressure that selects resistant bacteria and their accompanying genes. Given that these antibiotics have never been used for CDI treatment, the emergence of resistant *C. difficile* isolates could be explained by the acquisition of the responsible genes by other microorganisms sharing the same ecosystem.

The diversity of MGEs in the genome of *C. difficile* is closely associated with the dissemination of antimicrobial resistance. An in silico analysis revealed many MGEs and a variety of antimicrobial-resistance genes harbored therein. These encode resistance to various antibiotics, and most of these genes were located on genetic elements capable of lateral gene transfer. The similarity of these elements to those from unrelated bacteria indicates that genetic exchange between disparate microorganisms can occur readily. This notion is not surprising, since the gastrointestinal niche is an ideal environment for DNA exchange because of the proximity of bacterium to one another. In vitro experiments have shown that these elements can disseminate via lateral gene transfer events, which provides evidence supporting the hypothesis.

Ultimately, the development of antibiotic resistance in *C. difficile* is alarming, first with regard to the possibility for treatment failure in cases of infection and second, because of the potential to facilitate the transfer of resistance genes to other pathogens.

## Figures and Tables

**Table 1:** Frequency of mobile genetic elements in 1101 genomes of *C. difficile* available in the GenBank database ^1,2^.

Mobile Genetic Element	Genomes in Which MGEs Were Identified (n)	Genomes with MGEs in Which at Least One AMR Gene Was Identified (n)
Tn*6009*	408	71
Tn*6105*	269	31
CTn7	248	85
Tn*6192*	214	94
Tn*6194*	108	104
IS*256*	93	35
CTn4	38	13
Tn*6106*	31	10
CTn1	21	10
Tn*6218*	21	21
CTn6	21	5
CTn5	20	8
Tn*4453*	13	13
Tn*5397*	9	9
Tn*6073*	9	3
CTn2	6	0
Tn*6215*	6	6
Tn*5801* (B6)	5	5
MTnSag1	4	2
Tn*6164*	4	4
ISMlu*7*	3	0
Tn*5398*	3	3
Tn*6104*	2	0
IS*5*	1	0
ISAba*34*	1	0
ISClte*3*	1	0
ISEc*36*	1	0
ISEfa*9*	1	0
ISRgn*1*	1	0
ISRgn*2*	1	0
Tn*6107*	1	0
Tn*6263*	1	1

^1^ A total of 2190 genomes were evaluated, among which, in 1101, mobile genetic elements were found; ^2^ details of all abbreviations in Appendix A.

**Table 2:** Proportion of antimicrobial-resistance genes harbored in mobile genetic elements in the 1101 genomes of *C. difficile* available in the GenBank database ^1^.

Antimicrobial-Resistance Gene	Proportion among All Genes
*erm*(B)	17.03%
*blaCDD*	12.20%
*vanT*	11.32%
*vanR*	11.21%
*vanG*	10.88%
*vanS*	10.77%
*vanZ1*	5.82%
*aac(6′)-Ie/aph(2″)-Ia*	4.84%
*tet*(M)	4.29%
*cat*(P)	1.87%
*ant(6)-la*	1.65%
*blaCDD-*2	1.65%
*aph(3* *″* *)-IIIa*	1.10%
*sat4*	1.10%
*cfr*(C)	0.88%
*aadE*	0.55%
*spw*	0.55%
*tet*(44)	0.55%
*ant(6)-lb*	0.44%
*cfr*(B)	0.33%
*dfrF*	0.22%
*lnu*(C)	0.22%
*nmpA*	0.22%
*aad9*	0.11%
*aph(2″)-lf*	0.11%
*blaCDD*-1	0.11%

^1^ A total of 2190 genomes were evaluated, among which, in 1101, mobile genetic elements were found.

**Table 3:** Antimicrobial-resistance genes harbored in mobile genetic elements ^1^.

MGE ^2^	Genomes with MGEs in Which at Least One AMR Gene Was Identified (n) ^2^	MGEs in Which Antimicrobial-Resistance Genes Were Harbored (n)								
		*erm(B)*	*blaCDD*	*vanT*	*vanR*	*vanG*	*vanS*	*vanZ1*	*aac(6′)-Ie/aph(2″)-Ia*	*tet(M)*
Tn*6194*	104	104	23	4	4	4	4	15		7
Tn*6192*	94	5	29	27	28	27	27	8		4
CTn*7*	85	1	51	27	29	28	27	6		4
Tn*6009*	71	4	4	4		1	1	1	2	4
IS*256*	35	3	1	12	3		2	13	15	
Tn*6105*	31	1	3	6	18	16	17			4

^1^ The six MGEs most frequently identified as harbored AMR genes, and the nine AMR genes most frequently harbored therein are included. ^2^ Details of all abbreviations in Appendix A.

## Data Availability

All data are publicly available in the GenBank database.

## Appendix A

List of terms used as abbreviations in the manuscript.

**Abbreviation**	**Full Name of Term**
AMR	antimicrobial resistance
CDI	*Clostridioides difficile* infection
CRISPR	clustered regularly interspersed short palindromic repeat
CTn	conjugative transposon
DNA	deoxyribonucleic acid
ECE	extra-chromosomal element
GTP	guanosine triphosphate
ICE	integrative and conjugative elements
IS	insertion sequence
MGE	mobile genetic element
MLSB	macrolide-lincosamide and streptogramin B
MTn	multi-purpose transposon
PCR	polymerase chain reaction
PMN	polymorphonuclear neutrophils
RNA	ribonucleic acid
Tn	transposon
TndX	conjugative transposon site-specific recombinase
WGS	whole-genome sequencing
